# Curcumin Analogue CA15 Exhibits Anticancer Effects on HEp-2 Cells via Targeting NF-*κ*B

**DOI:** 10.1155/2017/4751260

**Published:** 2017-03-20

**Authors:** Jian Chen, Linlin Zhang, Yilai Shu, Liping Chen, Min Zhu, Song Yao, Jiabing Wang, Jianzhang Wu, Guang Liang, Haitao Wu, Wulan Li

**Affiliations:** ^1^Departments of Otolaryngology-Head and Neck Surgery, Eye, Ear, Nose and Throat Hospital, Fudan University, Shanghai, China; ^2^Chemical Biology Research Center, College of Pharmaceutical Sciences, Wenzhou Medical University, Wenzhou, Zhejiang, China; ^3^Departments of Stomatology, The First Affiliated Hospital of Wenzhou Medical University, Wenzhou, Zhejiang, China; ^4^College of Information Science and Computer Engineering, School of the first Clinical Medical Sciences, Wenzhou Medical University, Wenzhou, Zhejiang, China

## Abstract

Laryngeal carcinoma remains one of the most common malignancies, and curcumin has been proven to be effective against head and neck cancers in vitro. However, it has not yet been applied in clinical settings due to its low stability. In the current study, we synthesized 34 monocarbonyl analogues of curcumin with stable structures. CA15, which exhibited a stronger inhibited effect on laryngeal cancer cells HEp-2 but a lower toxicity on hepatic cells HL-7702 in MTT assay, was selected for further analysis. The effects of CA15 on cell viability, proliferation, migration, apoptosis, and NF-*κ*B activation were measured using MTT, Transwell migration, flow cytometry, Western blot, and immunofluorescence assays in HEp-2 cells. An NF-*κ*B inhibitor, BMS-345541, as well as curcumin was also tested. Results showed that CA15 induced decreased toxicity towards HL-7702 cells compared to curcumin and BMS-345541. However, similar to BMS-345541 and curcumin, CA15 not only significantly inhibited proliferation and migration and induced caspase-3-dependent apoptosis but also attenuated TNF-*α*-induced NF-*κ*B activation in HEp-2 cells. These results demonstrated that curcumin analogue CA15 exhibited anticancer effects on laryngeal cancer cells via targeting of NF-*κ*B.

## 1. Introduction

Laryngeal carcinoma remains one of the most common malignancies of human beings. Laryngeal squamous cell carcinoma (LSCC) comprises more than 95% of laryngeal cancers whose morbidity reaches 4.1/100,000 worldwide [1, 2]. Chemotherapy is considered as the most effective treatment for LSCC besides surgery, and cis-platinum which prominently improves the survival rate of LSCC patients is widely used [3, 4]. However, many side effects of chemotherapy have been reported, including leukopenia and kidney failure [5]. Exploitation of new drugs showing greater therapeutic efficacy but relatively low toxicity is of great interest nowadays.

Nuclear factor of *κ*B (NF-*κ*B) is a transcription factor which can bind to specific sequence known as *κ*B site [6]. It remains inactive by the inhibitor of *κ*B (I*κ*B) in the cytosol and could be activated by various carcinogenic factors including tumor necrosis factor-*α* (TNF-*α*) in cancer cells. The activation of myeloid differentiation protein 2 (MD2), a surface receptor of this signal, can induce the phosphorylation of I*κ*B kinase (IKK). When I*κ*B becomes phosphorylated, it dissociates from NF-*κ*B, thus enabling NF-*κ*B to translocate to the nucleus, bind to the *κ*B site, and activate genes to prompt cancer development afterwards [7]. NF-*κ*B is a potential target for cancer therapy.

Curcumin (diferuloylmethane), the major component isolated from the rhizomes of* Curcuma longa*, works as an anticancer agent in current researches [8]. Curcumin has shown impressive toxicity to LSCC in vitro and its inhibited effect on transcription factors NF-*κ*B may contribute at least partly to its anticancer effect [9–12]. However, its low stability and poor bioavailability in vivo motivate the modification of the molecule to acquire improved potency and physical properties [13]. It has been demonstrated that the *β*-diketone moiety probably results in the instability and rapid metabolism of curcumin [14].

Therefore, the monocarbonyl analogues of the curcumin (MCACs) which acquire a preferable stability were designed and synthesized in the present study. We carried out docking simulation of them with MD2 protein and screened out a promising compound named CA15 which exhibited a more notable effect against laryngeal cancer cells but a lower toxicity towards normal hepatic cells in comparison with curcumin. Furthermore, we investigated its anticancer effects against HEp-2 cells in detail and demonstrated that the agent could suppress NF-*κ*B signal by inhibiting phosphorylation of IKK in HEp-2 cells.

## 2. Materials and Methods

### 2.1. Cell Culture

Human laryngeal squamous cell carcinoma HEp-2 cells and human hepatic cells HL-7702 were obtained from the Cell Bank of the Chinese Academy of Sciences (Shanghai, China). They were cultured in RPMI-1640 (Gibco) medium with 10% fetal bovine serum (FBS, Gibco) and 1% penicillin/streptomycin in a humidified atmosphere at 37°C with 5% CO_2_. The medium was replaced every other day and cells in the following experiments were collected at the logarithmic growth phase.

### 2.2. Designs and Synthesis of MCACs

**Figure 1:**
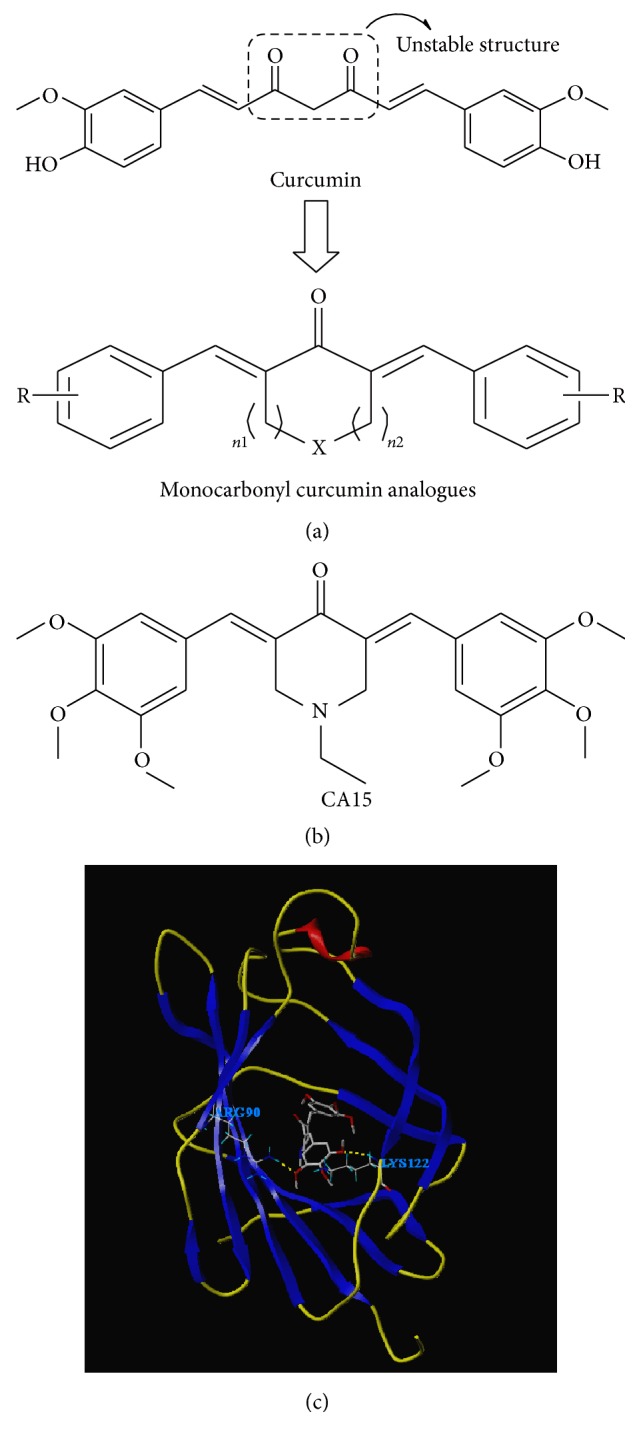
Design and synthesis of MCACs (a), chemical structure of curcumin analogue CA15 (b), and molecular docking of CA15 with MD2 (PDB ID 2E56). MCACs: monocarbonyl analogues of the curcumin.

Chemical constructions of curcumin and MCACs were depicted in Figure 1(a). A total of 34 MCACs were designed and synthesized by Chemical Biology Research Center, School of Pharmaceutical Sciences, Wenzhou Medical University, Wenzhou, Zhejiang, China. Supplementary Figure (in Supplementary Material available online at https://doi.org/10.1155/2017/4751260) shows their chemical constructions. All the compounds were dissolved in dimethyl sulfoxide (DMSO) and stored at −20°C before being diluted into final concentration by the culture medium in the following experiments.

### 2.3. Docking of MCACs to the MD2 Structural Model

The crystal structure of the MD2 was retrieved from Protein Data Bank (PDB ID: 2E56). SYBYL X-2.0 software was used for the preparation of protein and compounds. The target protein was prepared through extracting ligand structure, removing water molecular, adding charge in termini treatment, and adding hydrogen. Adding hydrogen, adding charge, Powell energy gradient method, Tripos force field, and Gasteiger-Hückel system were used to minimize the MCACs.

### 2.4. Cell Viability Assay

HEp-2 and HL-7702 cells were plated in a density of 5000 cells/well in 100 ul medium containing FBS and indicated test compounds were added. After 72 h incubation, the cell viability was evaluated by MTT assay and repeated at least three times. Briefly, each well was added with 20 *μ*l MTT solutions and then incubated for 4 h at 37°C in the dark. After removing the medium, 150 *μ*l DMSO was added to each well. The absorbance was measured using an ELISA plate reader at 490 nm. Half-maximal inhibitory concentrations (IC_50_) were determined using Sigma Plot 9.0 software (Systat Software Inc., CA) using the 4-parameter logistic function standard curve analysis for dose response.

### 2.5. Colony Formation Assay

Approximately 500 HEp-2 cells were cultured in medium containing 10% FBS at a final volume of 1 ml. Then the indicated compounds were added to the medium which was replaced 24 h later. After incubating for 7 days, cells were washed with PBS and fixed with 4% paraformaldehyde. The colonies were stained with crystal violet (Beyotime; Beijing, China) and photographed by a camera (Panasonic, Japan).

### 2.6. Transwell Migration Assay

Transwell migration chambers (8 *µ*m pore size; BD Biosciences, USA) were purchased for observing the chemotactic motility of cells. Firstly, each top chamber was filled with 200 *μ*L serum-free medium containing cells, while the bottom chamber was filled with 600 *μ*L RMPI-1640 medium containing 10% FBS. The indicated compounds were then added to both chambers at the same concentration. After a 24 h incubation in a humidified atmosphere, nonmigrated cells were erased by cotton swabs. The migrated cells were fixed with 4% paraformaldehyde and stained with crystal violet solution. Images were photographed using an inverted microscope (Nikon; Japan) and the cells in at least 3 random microscopic fields were counted.

### 2.7. Measurements of Apoptosis

Approximately 1 million HEp-2 cells were incubated with indicated compounds for 12 h. Subsequently, cells were washed with cold PBS, harvested in binding buffer, and successively incubated with 3 *µ*l Annexin V-FITC and 1 *µ*l of PI (BD Biosciences, San Jose, CA, USA) for 15 min at room temperature in darkness. Apoptosis was determined by a Flow Cytometer (BD Biosciences, USA). For flow cytometric dot plot, Annexin V staining was set as the horizontal axis and PI staining was set as the vertical axis. Early apoptosis cells in the lower right quadrant and late apoptosis or necrotic cells in the upper right quadrant of the flow cytometric dot plot were both calculated.

### 2.8. Western Blot Analysis

Cells that needed examination were lysed in RIPA buffer to extract the total cellular protein. Protein concentration was determined and 60 *µ*g of each protein sample was boiled at 100°C in SDS sample buffer for 10 min, electrophoresed on 10% SDS/PAGE gels, and then transferred to polyvinylidene difluoride (PVDF) membranes. Following protein transfer, the membranes were blocked by 5% skimmed milk proteins for 90 min, followed by incubation at 4°C overnight with the primary antibodies. Membranes were incubated with correlate secondary antibody at room temperature for 1 h on the second day, and specific protein bands were detected with an enhanced chemiluminescence (ECL) assay kit (BD, USA). The primary antibodies used were as follows: Cle-PARP, Bcl-2, Bax, p-IKK, IKK, I*κ*B-*α*, and GAPDH (Santa Cruz Biotechnology, USA) and Cle-caspase-3 (Cell Signaling Technology, USA).

### 2.9. Immunofluorescence

Approximately 500,000 HEp-2 cells were seeded on every glass slide in the 6-well plate and incubated with indicated compounds for 1 h. Cells were fixed in 4% paraformaldehyde for 15 min and permeated by 0.3% Triton X-100 (Beyotime; Beijing, China) for another 15 min. After blocking with 10% goat serum, slides were washed and incubated with anti-NF-*κ*B-p65 antibody (Santa Cruz Biotechnology, USA) at 4°C overnight. Afterwards, they were incubated with PE-conjugated secondary antibody (Santa Cruz Biotechnology, USA) for 1 h at room temperature in the dark on the following day. After PBS washing, the slides were counterstained using 5 *µ*g/ml DAPI solutions (Sigma, USA) for 5 min before being photographed by a fluorescent microscope (Nikon, Japan).

### 2.10. Statistical Analysis

All results are expressed as means ± standard errors from three independent experiments. The statistical analysis was performed by using Student's *t*-test or one-way analysis of variance (GraphPad Prism 5.0). *P* < 0.05 was considered to be statistically significant.

## 3. Results

### 3.1. CA15 Acted with MD2 Protein on Arg^90^ and Lys^122^ Residues and Achieved the Highest Total Score

The molecular docking experiments were performed at PH 7.0. As shown in Table 1, only 7 of these compounds had acting sites with MD2 protein using a molecular simulation. CA15 had the highest total score among 7 agents and might be the most probable candidate as an anticancer agent of NF-*κ*B.  Figure 1(b) depicted its chemical structure, and the computer-assisted simulation indicated that Arg^90^ and Lys^122^, two amino residues of MD2 protein, were most likely to form hydrogen bonds with CA15 (Figure 1(c)).

### 3.2. CA15 Exhibited a More Preferable Cytotoxicity to HEp-2 Cells but a Lower Toxicity to HL-7702 Cells

The inhibitory rates of MCACs on HEp-2 and HL-7702 cells at the concentration of 20 *μ*M were shown in Supplementary Table. CA15 was selected for further analysis, which expressed an inhibitory rate of 83.38 ± 4.79% on HEp-2 cells while only 36.51 ± 3.21% on HL-7702 cells at the concentration of 20 *μ*M. Furthermore, IC_50_ values of CA15 were also tested by MTT. A highly selective inhibitor of NF-*κ*B, BMS-345541 (BMS, Sigma) [15], was used as control as well as curcumin and its well-known analogues (EF24 and B19). Though without a significant difference, CA15 exhibited a more preferable cytotoxicity to HEp-2 cells but a lower toxicity to normal cells HL-7702 when compared to curcumin (Table 2). Stronger toxic effect on HL-7702 cells was observed in BMS compared to curcumin, which had the lowest IC_50_ value on HEp-2 cells (Table 2).

### 3.3. CA15 Inhibited Proliferation and Migration of HEp-2 Cells

**Figure 2:**
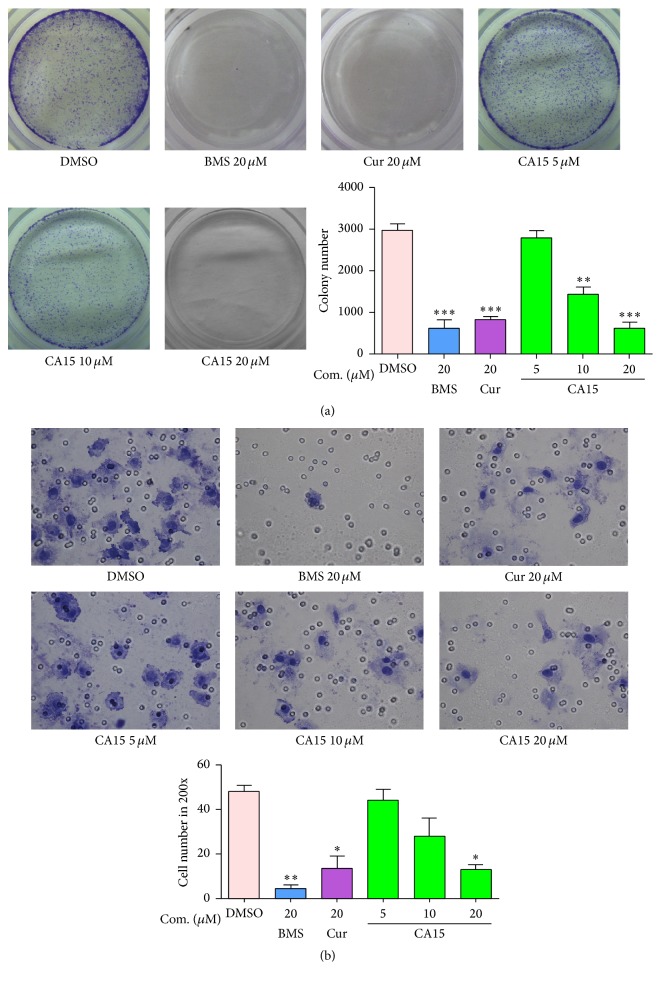
CA15 suppressed proliferation and migration in HEp-2 cells. (a) HEp-2 cells were treated with different concentrations (0–20 *μ*M) of CA15, 20 *μ*M Cur, and 20 *μ*M BMS for 24 h. Cell proliferation was evaluated with colony formation assay. (b) HEp-2 cells were treated with different concentrations (0–20 *μ*M) of CA15, 20 *μ*M Cur, and 20 *μ*M BMS for 24 h. Cell migration was examined using Transwell migration chambers and then observed under a microscope (magnification ×200). The graph displays means ± SEM of 3 independent experiments. ^*∗*^*P* < 0.05, ^*∗∗*^*P* < 0.01, and ^*∗∗∗*^*P* < 0.001 versus DMSO group. Cur: curcumin; BMS: BMS-345541.

To investigate the anticancer effects of CA15 on HEp-2 cells in detail, its inhibitory effect on colony formation of HEp-2 cells was examined firstly. As shown in Figure 2(a), CA15 suppressed colony formations of HEp-2 cells in a dose-dependent manner. By Transwell assay, we found that HEp-2 cells treated with curcumin or BMS showed obviously inhibited migration capability. Similarly, CA15 inhibited transmigration of HEp-2 significantly at the concentration of 20 *μ*M (*P* < 0.01, Figure 2(b)).

### 3.4. CA15 Induced Apoptosis via Bax/Bcl-2 and Caspase-3-Dependent Pathway in HEp-2 Cells

**Figure 3:**
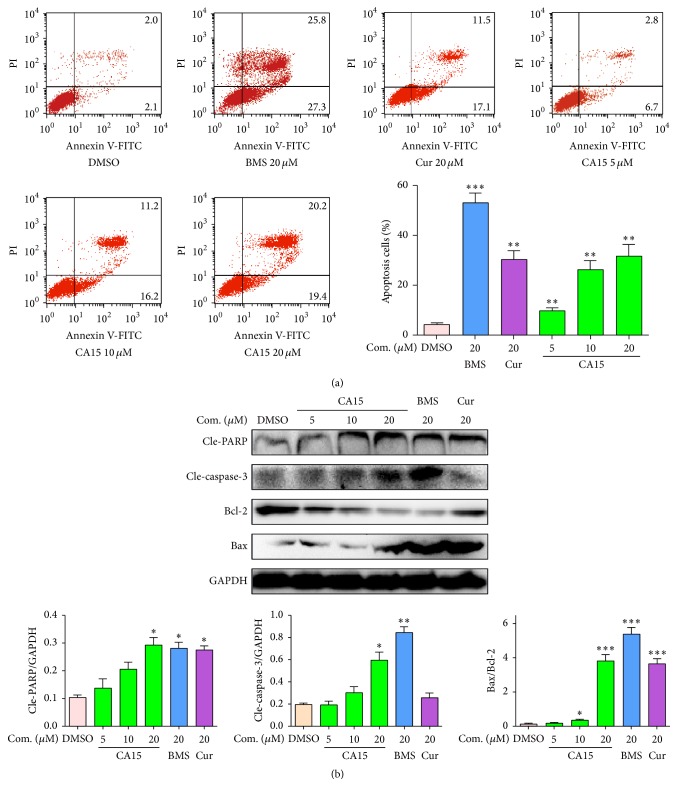
CA15 induced apoptosis by upregulating Bax/Bcl-2 ratio followed by caspase-3 cleavage in HEp-2 cells. HEp-2 cells were treated with different concentrations (0–20 *μ*M) of CA15, 20 *μ*M Cur, and 20 *μ*M BMS for 24 h and analyzed by flow cytometry (a) or for 12 h and detected by Western blot (b). The graphs display means ± SEM of 3 independent experiments. ^*∗*^*P* < 0.05, ^*∗∗*^*P* < 0.01, and ^*∗∗∗*^*P* < 0.001 versus DMSO group. Cur: curcumin; BMS: BMS-345541.

We observed that CA15 could induce apoptosis in HEp-2 cells using Annexin V/PI. Apoptotic rates of HEp-2 cells treated with 5 *μ*M CA15, 10 *μ*M CA15, and 20 *μ*M CA15 were 9.78 ± 0.93%, 26.18 ± 3.72%, and 31.53 ± 4.76%, respectively, which were all significantly increased compared to the control group (Figure 3(a)). Western blot analysis revealed that CA15 decreased the antiapoptotic Bcl-2 protein, while it increased the proapoptotic Bax in a dose-dependent manner (Figure 3(b)). As expected, elevated Bax/Bcl-2 ratio led to cleavage and activation of PARP and caspase-3 (two markers for cell apoptosis) in HEp-2 cells (Figure 3(b)). Collectively, these results suggested that CA15 resulted in apoptosis induction via Bax/Bcl-2 and caspase-3-dependent pathway.

### 3.5. CA15 Inhibited TNF-*α*-Induced NF-*κ*B Activation in HEp-2 Cells

**Figure 4:**
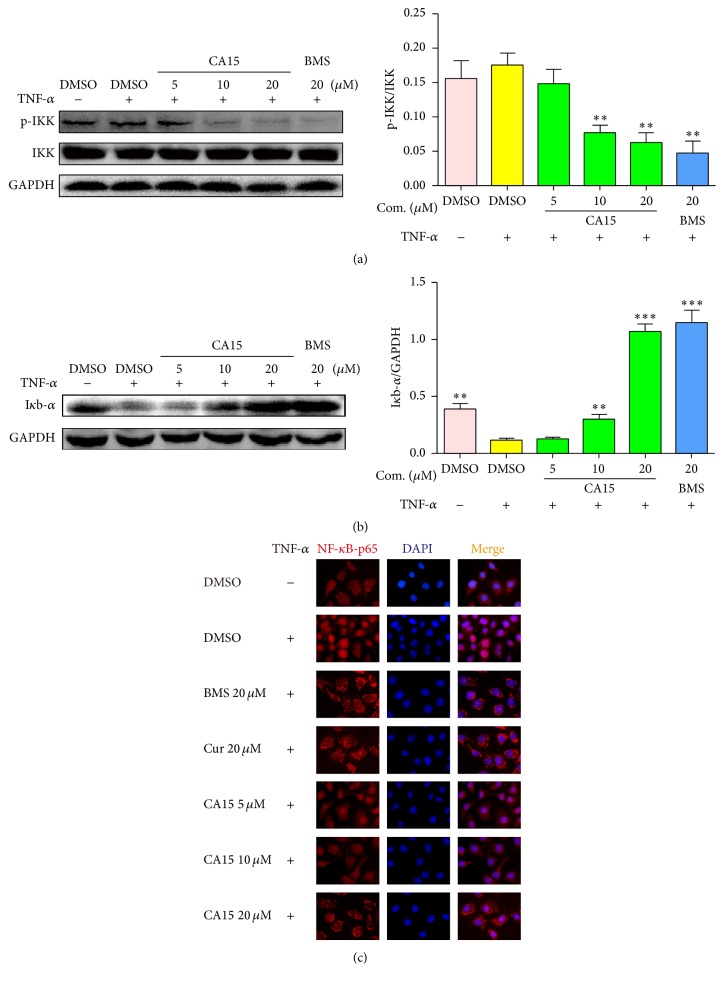
CA15 inhibited TNF-*α*-induced NF-*κ*B activation in HEp-2 cells. (a and b) HEp-2 cells were pretreated with different concentrations (0–20 *μ*M) of CA15 and 20 *μ*M BMS for 1 h followed by incubation with TNF-*α* (5 ng/ml) for 15 min (a) or 30 min (b). The protein levels of p-IKK, IKK, and I*κ*B were examined by Western blot. The graphs display means ± SEM of 3 independent experiments. ^*∗∗*^*P* < 0.01 and ^*∗∗∗*^*P* < 0.001 versus TNF-*α* treated group. (c) HEp-2 cells were pretreated with 20 *μ*M CA15 and 20 *μ*M BMS for 1 h followed by incubation with TNF-*α* (5 ng/ml) for 60 min. Cells were then incubated with NF-*κ*B-p65 antibody and PE-conjugated secondary antibody (red), and the nuclei were stained with DAPI (blue). The photographs were obtained by fluorescence microscope (magnification ×200). TNF-*α*: tumor necrosis factor-*α*; Cur: curcumin; BMS: BMS-345541.

Finally, whether CA15 could inhibit NF-*κ*B activation in HEp-2 cells was investigated. As shown in Figure 4(a), TNF-*α*-induced phosphorylation of IKK was strikingly decreased in a dose-dependent manner following pretreatment with CA15. Phosphorylation of IKK leads to degradation of I*κ*B. As expected, pretreatment with CA15 markedly reversed TNF-*α*-induced I*κ*B degradation in HEp-2 cells in a dose-dependent manner (Figure 4(b)). NF-*κ*B-p65 is able to translocate to the nucleus and raise its DNA-binding capacity without inhibition of I*κ*B. Consequently, TNF-*α* vastly prompted the nuclear translocation of NF-*κ*B-p65, whereas CA15 suppressed this process significantly apart from curcumin and BMS (Figure 4(c)).

## 4. Discussion

Although treatments of LSCC with surgery and chemoradiotherapy have been improved vastly in recent years [16, 17], the unintentional injuries on laryngeal functions and quality of life remain inevitable. As a natural compound isolated from plants, curcumin has a low toxicity but a remarkable anticancer effect on various malignancies such as lung cancer, liver cancer, and colon cancer [18–20]. Apart from that, curcumin is considered to be a repressor of LSCC growth via topical application [9]. However, the usage of curcumin is limited by the poor bioavailability due to its *β*-diketonate structure [13]. Although novel biocompatible nanosystems for curcumin delivery have been developed, the anticancer effects of curcumin nanoparticles could not last for a long time [21, 22]. The *β*-diketonate structure in curcumin is modified in MCACs (Figure 1(a)), which could improve its chemical stability in a different way [23].

Therefore, we synthesized 34 MCACs and screened out CA15 which performed a stronger inhibitory effect on laryngeal cancer cells HEp-2 but a lower toxicity on hepatic cells HL-7702 compared to curcumin (Table 2). A number of MCACs have been reported to have a similar biological effect but a more stable chemical structure compared to curcumin [23–26]. As a curcumin analogue with greater bioavailability and biological activity, EF24 is able to inhibit proliferation of colon cancer cells and migratory of melanoma cells [27, 28]. Another curcumin analogue, B19, is also found to exert antiangiogenic activity and induce apoptosis of ovarian cancer cells [29, 30]. However, their toxicities towards normal cells are always neglected. They were both highly toxic to normal hepatic cells in our study. By contrast, CA15 treated HL-7702 cells that presented higher IC_50_ value, which indicated that CA15 might be a less toxic drug compared to curcumin (Table 2). Nevertheless, CA15, just like curcumin, was able to trigger caspase-3-dependent apoptosis in HEp-2 cells and exhibit remarkable inhibition on cell proliferation and migration.

As we know, whether a cell goes into the programmed cell death partly depends on the balance between proteins that promote cell viability (e.g., Bcl-2) and proteins that mediate apoptosis (e.g., PARP and Bax). Downregulation of Bcl-2 upregulates Bax and increases the Bax/Bcl-2 ratio, thereby inducing cleavage of PARP and caspase-3 and ultimately promoting hydrolysis of cytoskeletal proteins and nucleic acids [31]. NF-*κ*B is a key transcription factor that targets Bcl-2 and regulates caspase-3-dependent apoptosis [32]. It has been reported that NF-*κ*B activated cells exhibited enhanced expression of Bcl-2 protein, while NF-*κ*B inhibition impaired Bcl-2 expression [33]. The dysregulation or chronic activation of NF-*κ*B signaling restrains the programmed cell death of cancer cells, thus contributing a lot to the occurrence of many cancers including head and neck squamous cell carcinoma (HNSCC). A study reported that over 1,000 NF-*κ*B targeting genes are differentially expressed in head and neck squamous cell carcinoma (HNSCC) in comparison with nonmalignant keratinocytes, along with the aberrant activation of NF-*κ*B signaling [34]. These known genes are involved in the process of proliferation, diversification, angiogenesis, adhesion, and epithelial-mesenchymal transition of cancer cells, thus assisting tumors in evasion of apoptosis, sustained growth, invasion, and metastasis [34]. A number of factors may contribute to the activation of NF-*κ*B in HNSCC, such as stimuli of tobacco and alcohol and infections of EB and HPV [35–37]. Activation of NF-*κ*B prompts the invasion and metastasis process of HNSCC [38, 39] and is probably associated with poor outcome among these patients [39–41]. Accordantly, the increase of NF-*κ*B associated cytokines, such as IL-6 and VEGF, may predict a poor prognosis in HNSCC patients [42–44]. Furthermore, cancer cells with defective NF-*κ*B signaling seemed to be more sensitive to chemotherapy, which suggests that NF-*κ*B should be probably involved in drug-resistance of HNSCC [45]. Taken together, NF-*κ*B may promote cancer occurrence, progression, and drug resistance in HNSCC.

Currently, therapeutics based on targeting NF-*κ*B are of considerable interest. Our study also demonstrated that CA15 could inhibit NF-*κ*B signaling in HEp-2 cells. The mechanism of CA15 targeting NF-*κ*B is like BMS, which acts as a highly selective inhibitor of IKK which binds at an allosteric site of the enzyme [15]. CA15 can inhibit the phosphorylation of IKK and hinder the degradation of I*κ*B which binds to NF-*κ*B-p65, thereby preventing NF-*κ*B-p65 from translocating to the nucleus and regulating related genes (Figure 4). Several experiments demonstrated that intervention of NF-*κ*B signal is a potential approach to cure LSCC [46, 47]. Agents such as PDTC, celecoxib, guggulsterone, and bortezomib have already showed impressive inhibitory effects on HNSCC via targeting NF-*κ*B [48–51]. NF-*κ*B might be a major focus of therapeutic intervention for LSCC treatments.

However, there is still limited knowledge regarding the specific mechanisms of CA15 against cancers. Also, its toxicity in vivo was not tested in our study. Further researches into the mechanisms and toxicity in vivo are warranted.

## 5. Conclusion

The current study reveals that CA15, a novel monocarbonyl curcumin analogue, exhibits preferable anticancer effects via targeting NF-*κ*B but little toxicity to normal cells. NF-*κ*B may be a potential target for LSCC treatment, and CA15, perhaps, has a potential therapeutic use in the treatment of laryngeal cancer in the future.

## Supplementary Material

Supplementary Table: HEp-2 and HL7702 cells were treated with these compounds at the concentration of 20 μM for 72 h and then tested with MTT assay. Data are presented as means ± SEM of 3 independent experiments. MCACs: Mono-carbonyl analogues of the curcumin.
Supplementary Figure: The chemical constructions of synthesized MCACs. MCACs: mono-carbonyl analogues of the curcumin.

## Figures and Tables

**Table 1:** Docking of MCACs to the MD2 structural model.

Compounds	Acting sites	Total score
CA15	ARG^90^, LYS^122^	7.38
CA32	SER^120^	6.40
CA33	SER^120^	6.02
CA1	TYR^102^	5.92
CA2	TYR^102^	5.61
CA34	LYS^122^	5.01
CA28	ARG^90^	4.41

MCACs: monocarbonyl analogues of the curcumin; MD2: myeloid differentiation protein 2.

**Table 2:** The IC_50_ values of compounds towards HEp-2 and HL-7702 cells.

Group	HEp-2 (*μ*M)	HL-7702 (*μ*M)
Curcumin	21.37 ± 3.25	32.33 ± 6.21
CA15	16.59 ± 2.43	46.79 ± 8.77
EF24	14.09 ± 5.32	12.87 ± 2.73^*∗*^
B19	11.69 ± 2.66	15.19 ± 2.76
BMS-345541	7.58 ± 0.41^*∗*^	23.63 ± 3.24

HEp-2 and HL-7702 cells were treated with different concentrations (0–60 *μ*M) of compounds for 72 h and then tested with MTT assay. Data are presented as means ± SEM of 3 independent experiments. ^*∗*^*P* < 0.05 versus curcumin group.
