# Corrigendum to “Curcumin Analogue CA15 Exhibits Anticancer Effects on HEp-2 Cells via Targeting NF-*κ*B”

**DOI:** 10.1155/2017/3236424

**Published:** 2017-12-28

**Authors:** Jian Chen, Linlin Zhang, Yilai Shu, Liping Chen, Min Zhu, Song Yao, Jiabing Wang, Jianzhang Wu, Guang Liang, Haitao Wu, Wulan Li

**Affiliations:** ^1^Departments of Otolaryngology-Head and Neck Surgery, Eye, Ear, Nose and Throat Hospital, Fudan University, Shanghai, China; ^2^Chemical Biology Research Center, College of Pharmaceutical Sciences, Wenzhou Medical University, Wenzhou, Zhejiang, China; ^3^Departments of Stomatology, The First Affiliated Hospital of Wenzhou Medical University, Wenzhou, Zhejiang, China; ^4^College of Information Science and Computer Engineering, School of the First Clinical Medical Sciences, Wenzhou Medical University, Wenzhou, Zhejiang, China

In the article titled “Curcumin Analogue CA15 Exhibits Anticancer Effects on HEp-2 Cells via Targeting NF-*κ*B” [1], the cell line HEp-2 used in the study had been reported to be contaminated by HeLa, a cervical cancer cell line. Therefore, the study can only prove that the compound CA15 has antitumor effects, not antihuman laryngeal carcinoma.
