# Supplementary material for: Curcumin Analogue CA15 Exhibits Anticancer Effects on HEp-2 Cells via Targeting NF-κB
Source: Biomed Res Int. 2017 Mar 20;2017:4751260. doi: 10.1155/2017/4751260 (PMC5376929; doi:10.1155/2017/4751260)
Supplement: Supplementary file 1 — Supplementary Table: HEp-2 and HL7702 cells were treated with these compounds at the concentration of 20 μM for 72 h and then tested with MTT assay. Data are presented as means ± SEM of 3 independent experiments. MCACs: Mono-carbonyl analogues of the curcumin. Supplementary Figure: The chemical constructions of synthesized MCACs. MCACs: mono-carbonyl analogues of the curcumin. [file 4751260.f1.docx]

**Supplementary Table.** The inhibitory rates of MCACs on hep-2 and HL7702 cells at the concentration of 20μM

| Compounds(20μM) | Hep-2(%) | HL7702(%) |
| --- | --- | --- |
| CA1 | 30.20±8.99 | 4.41±2.64 |
| CA2 | 31.13±7.40 | 7.49±0.24 |
| CA3 | 49.82±2.59 | 27.93±5.96 |
| CA4 | 83.47±4.01 | 64.76±6.70 |
| CA5 | 79.29±4.44 | 66.90±3.92 |
| CA6 | 29.86±2.16 | 11.32±2.93 |
| CA7 | 41.03±14.52 | 29.73±2.89 |
| CA8 | 78.49±6.91 | 56.30±6.76 |
| CA9 | 85.85±3.22 | 54.34±11.75 |
| CA10 | 81.71±3.26 | 55.53±7.80 |
| CA11 | 21.26±6.81 | 3.90±1.85 |
| CA12 | 81.40±1.88 | 55.98±6.67 |
| CA13 | 84.26±3.83 | 59.00 ±5.13 |
| CA14 | 84.63±2.21 | 60.43±12.12 |
| CA15 | 83.38±4.79 | 36.51±3.21 |
| CA16 | 80.09±1.63 | 58.28±9.02 |
| CA17 | 83.21±1.05 | 64.28±3.38 |
| CA18 | 4.85±2.38 | 16.67±1.43 |
| CA19 | 78.92±7.89 | 61.24±4.51 |
| CA20 | 60.40±11.16 | 57.98±10.40 |
| CA21 | 3.08±2.71 | 17.18±8.26 |
| CA22 | 74.19±5.04 | 59.51±6.26 |
| CA23 | 80.51±6.39 | 63.39±2.95 |
| CA24 | 61.48±3.02 | 62.78±2.90 |
| CA25 | 4.49±3.97 | 8.44±2.71 |
| CA26 | 7.15±4.60 | 42.66±10.65 |
| CA27 | 78.83±8.46 | 68.16±5.41 |
| CA28 | 86.37±0.75 | 54.21±5.40 |
| CA29 | 74.34±3.60 | 54.98±13.15 |
| CA30 | 85.28±3.72 | 64.04±11.28 |
| CA31 | 89.33±0.40 | 65.36±4.58 |
| CA32 | 92.23±5.98 | 64.99±16.47 |
| CA33 | 20.97±6.21 | 62.78±6.92 |
| CA34 | 12.92±7.86 | 1.38±0.43 |

HEp-2 and HL7702 cells were treated with these compounds at the concentration of 20μM for 72h and then tested with MTT assay. Data are presented as means ± SEM of 3 independent experiments. MCACs: Mono-carbonyl analogues of the curcumin
