# Supplementary figures and images for: Curcumin Analogue CA15 Exhibits Anticancer Effects on HEp-2 Cells via Targeting NF-κB
Source: Biomed Res Int. 2017 Mar 20;2017:4751260. doi: 10.1155/2017/4751260 (PMC5376929; doi:10.1155/2017/4751260)

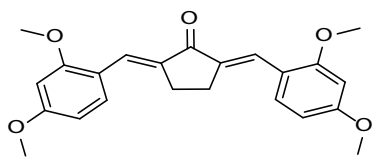

CA1

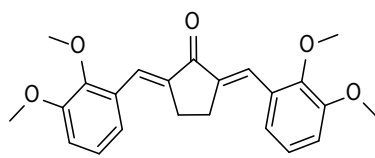

CA2

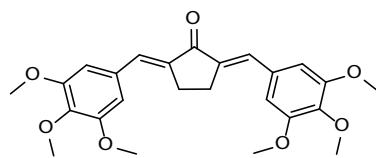

CA3

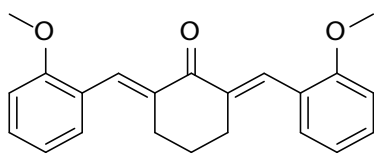

CA4

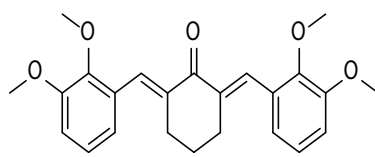

CA5

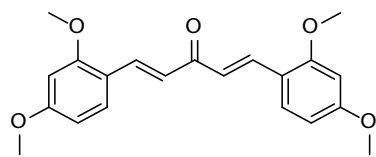

CA6

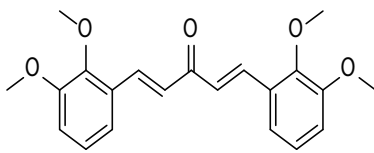

CA7

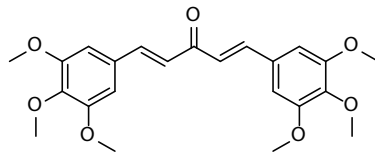

CA8

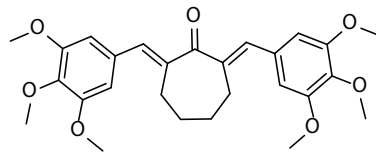

CA9

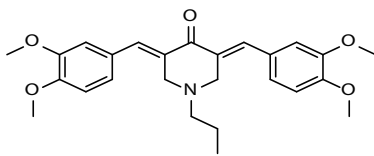

CA10

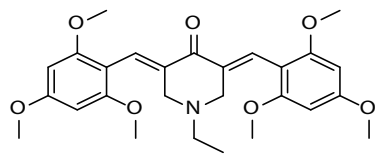

CA11

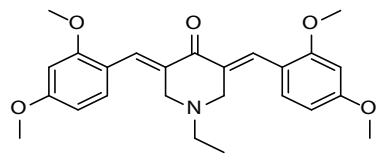

CA12

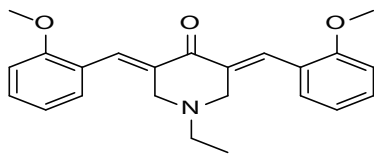

CA13

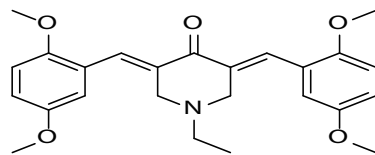

CA14

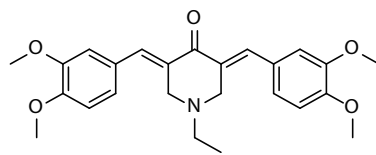

CA16

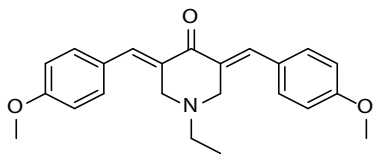

CA17

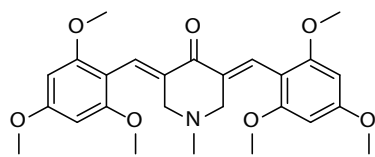

CA18

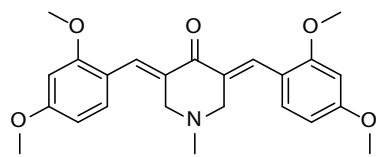

CA19

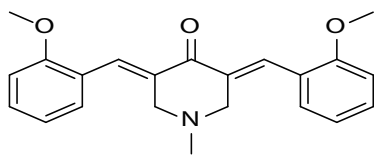

CA20

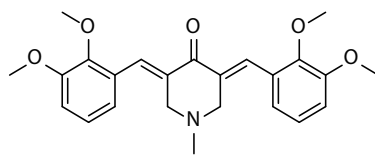

CA21

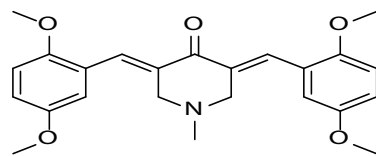

CA22

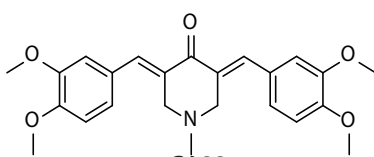

CA23

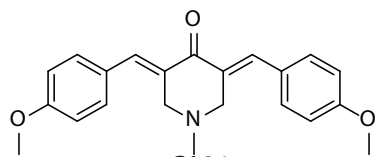

CA24

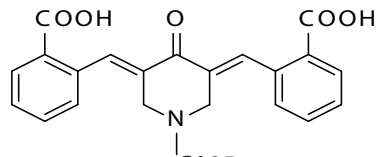

CA25

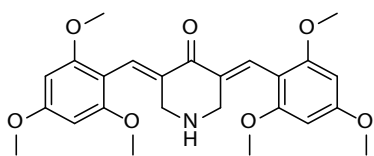

CA26

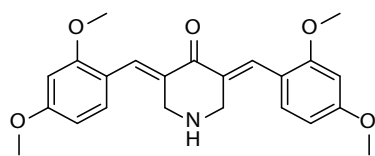

CA27

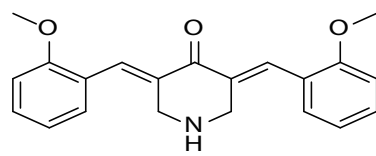

CA28

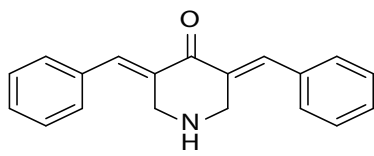

CA29

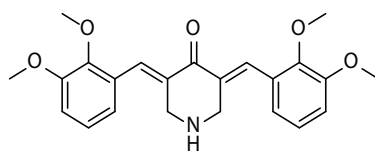

CA30

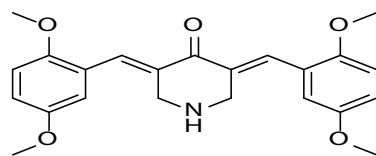

CA31

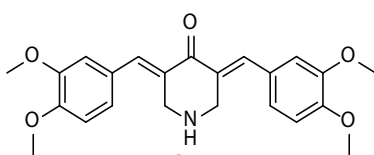

CA32

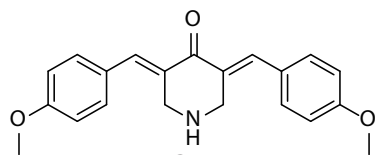

CA33

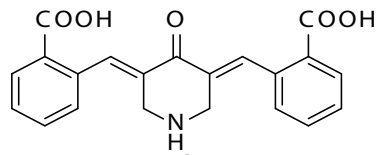

CA34

Supplement: Supplementary file 2 [file 4751260.f2.pdf]
